# Zwitterionic 4-bromo-6-meth­oxy-2-{[tris­(hy­droxy­meth­yl)methyl]­iminiumyl­meth­yl}phenolate: crystal structure and Hirshfeld surface analysis

**DOI:** 10.1107/S2056989016012159

**Published:** 2016-07-29

**Authors:** See Mun Lee, Kong Mun Lo, Sang Loon Tan, Edward R. T. Tiekink

**Affiliations:** a Research Centre for Crystalline Materials, Faculty of Science and Technology, Sunway University, 47500 Bandar Sunway, Selangor Darul Ehsan, Malaysia

**Keywords:** crystal structure, zwitterion, hydrogen bonding, Hirshfeld surface analysis

## Abstract

In the solid state, the title compound, C_12_H_16_BrNO_5_ [systematic name: 4-bromo-2-((1*E*)-{[1,3-dihy­droxy-2-(hy­droxy­meth­yl)propan-2-yl]iminium­yl}meth­yl)-6-meth­oxy­benzen-1-olate], C_12_H_16_BrNO_5_, is found in the keto–amine tautomeric form, with an intra­molecular iminium-N—H⋯O(phenolate) hydrogen bond and an *E* conformation about the C=N bond. Both *gauche* (two) and *anti* relationships are found for the methyl­hydroxy groups. In the crystal, a supra­molecular layer in the *bc* plane is formed *via* hy­droxy-O—H⋯O(hy­droxy) and charge-assisted hy­droxy-O—H⋯O(phenolate) hydrogen-bonding inter­actions; various C—H⋯O inter­actions provide additional cohesion to the layers, which stack along the *a* axis with no directional inter­actions between them. A Hirshfeld surface analysis confirms the lack of specific inter­actions in the inter-layer region.

## Abstract 2

The title compound exists in the keto–amine tautomeric form. In the crystal, supra­molecular layers are formed by O—H⋯O hydrogen bonding.

## Chemical context   

Inter­est in mol­ecules related to the title Schiff base compound derived from tris­(hy­droxy­meth­yl)amino­methane (see Scheme) rests largely with the biological activity exhibited by their metal complexes. Thus, various species have been studied for their anticancer potential, *e.g*. vanadium (Back *et al.*, 2012 ▸) and tin (Lee *et al.*, 2015 ▸). The insulin-mimetic behaviour of vanadium complexes have been explored (Rehder *et al.*, 2002 ▸), as has the catecolase activity of binuclear cobalt complexes (Dey & Mukherjee, 2014 ▸). More recently, the adipogenic (cell differentiation) capacity of vanadium (Halevas *et al.*, 2015 ▸) and zinc complexes has been described (Tsave *et al.*, 2015 ▸). Over and above these considerations, magnetochemistry motivates on-going investigations, especially single-mol­ecule (Wu *et al.*, 2007 ▸; Chandrasekhar *et al.*, 2013 ▸; Dey *et al.*, 2015 ▸) and lanthanide-containing species (Zou *et al.*, 2015 ▸; Das *et al.*, 2015 ▸). It was during on-going biological assays (Lee *et al.*, 2015 ▸) that the title compound, (I), became available. Herein, the crystal and mol­ecular structures of (I) are described, as well as a Hirshfeld surface analysis.

## Structural commentary   

**Figure 1:**
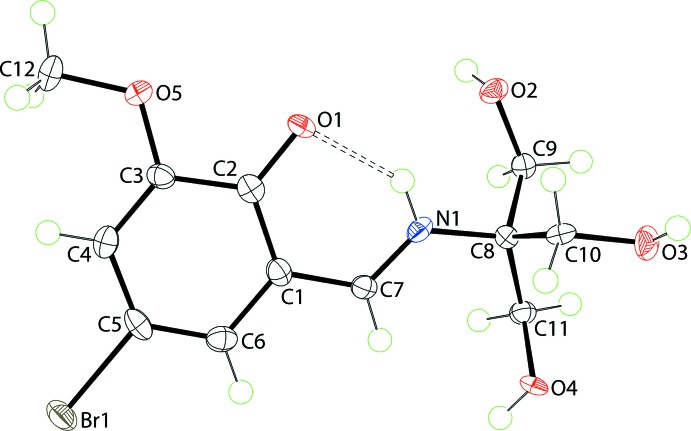
The mol­ecular structure of (I), showing the atom-labelling scheme and displacement ellipsoids at the 70% probability level. The intramolecular N—H⋯O hydrogen bond is shown as a double-dashed line (see Table 1 ▸)

The mol­ecular structure of (I) (Fig. 1 ▸) exists as a zwitterion in the solid state, with the iminium N atom being protonated and the phenolate O atom being deprotonated. The observed keto–amine tautomeric form for (I) is the common form for mol­ecules of this type, see *Database survey*. The conformation about the iminium bond [1.295 (4) Å] is *E* and this residue is almost coplanar with the benzene ring, forming a C2—C1—C7—N1 torsion angle of 1.9 (4)°. This arrangement allows for the formation of a tight charge-assisted iminium-N—H⋯O(phenolate) hydrogen bond (Table 1 ▸). The conformations of the methyl­hydroxy groups are variable, with *gauche* relationships about the C8—C9 and C8—C11 bonds [N1—C8—C9—O2 is 45.9 (3)°, *i.e*. +synclinal, and N1—C8—C11—O4 is −80.2 (3)°, *i.e*. –synclinal], and an *anti* relationship about the C8—C10 bond [N1—C8—C10—O3 is 178.8 (2)°, *i.e*. +anti­periplanar]. The meth­oxy group is almost coplanar with the ring it is connected to, as seen in the value of the C12—O5—C3—C2 torsion angle of 177.7 (2)°.
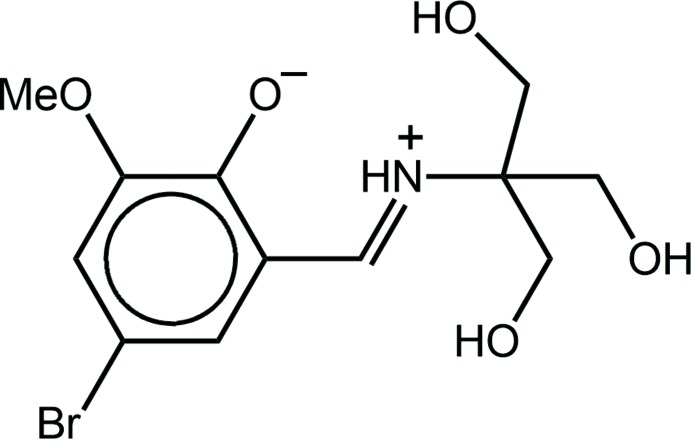


## Supra­molecular features   

**Figure 2:**
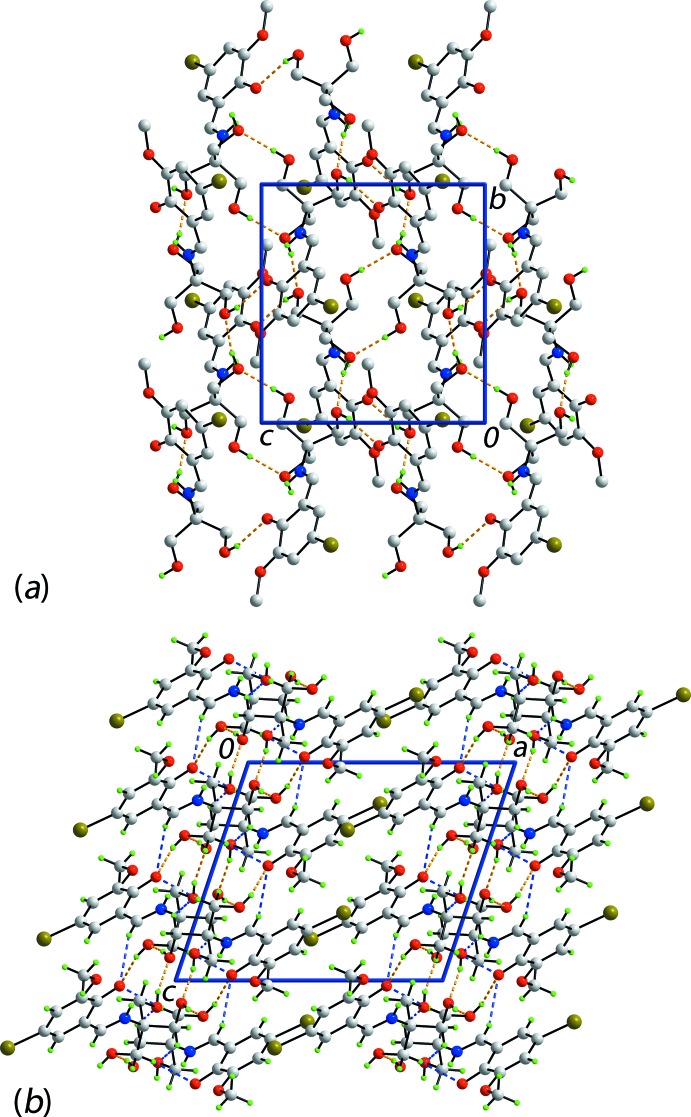
The mol­ecular packing in (I), showing (*a*) a view of the supra­molecular layer sustained by O—H⋯O hydrogen bonding, shown as orange dashed lines, and (*b*) a view of the unit-cell contents shown in projection down the *b* axis, highlighting the stacking of layers along the *a* axis. In (*a*), only acidic H atoms are shown.

As anti­cipated from the chemical composition of (I), there are considerable hydrogen-bonding inter­actions operating in the crystal; geometric characteristics of these are listed in Table 1 ▸. Each of the hy­droxy O2 and O3 atoms participates in hy­droxy-O—H⋯O(hy­droxy) hydrogen-bonding inter­actions, while the hy­droxy O4 atom forms a donor inter­action with the phenolate O1 atom. The result is the formation of a supra­molecular layer parallel to (100) (Fig. 2 ▸ *a*). Within this framework are a number of C—H⋯O inter­actions, *i.e*. imine-C7—H⋯O(phenolate), methyl­ene-C11—H⋯O(phenolate) and methyl­ene-C9—H⋯O(hy­droxy) (Fig. 2 ▸ *b*). In accord with the distance criteria in *PLATON* (Spek, 2009 ▸), layers stack along the *a* axis with no directional inter­actions between them. In order to gain more insight into the mol­ecular packing of (I), a Hirshfeld surface analysis was conducted.

## Analysis of the Hirshfeld surfaces   

The Hirshfeld surface of (I) was mapped over the *d*_norm_ contact distance within the range of −0.67 to 1.31 Å through calculation of the inter­nal (*d*_i_) and external (*d*_e_) Hirshfeld surface distances to the nearest nucleus (McKinnon *et al.*, 2007 ▸; Spackman & Jayatilaka, 2009 ▸). Two-dimensional fingerprint plots associated with relevant close contacts were obtained through the plot of *d*_e_ *versus d*_i_ (Spackman & McKinnon, 2002 ▸). The electrostatic potential (ESP) of the crystal structure was mapped onto the Hirshfeld surface by an *ab initio* quantum modelling approach at the Hartree–Fock level of theory with the STO-3G basis set (HF/STO-3G) over the range of −0.122 to 0.189 au. All Hirshfeld surface and fingerprints plots were generated using *Crystal Explorer* (Wolff *et al.*, 2012 ▸), while the ESP was calculated by *TONTO* (Spackman *et al.*, 2008 ▸) as implemented in *Crystal Explorer*. Distances involving H atoms were normalized to the standard neutron diffraction bond lengths.

**Figure 3:**
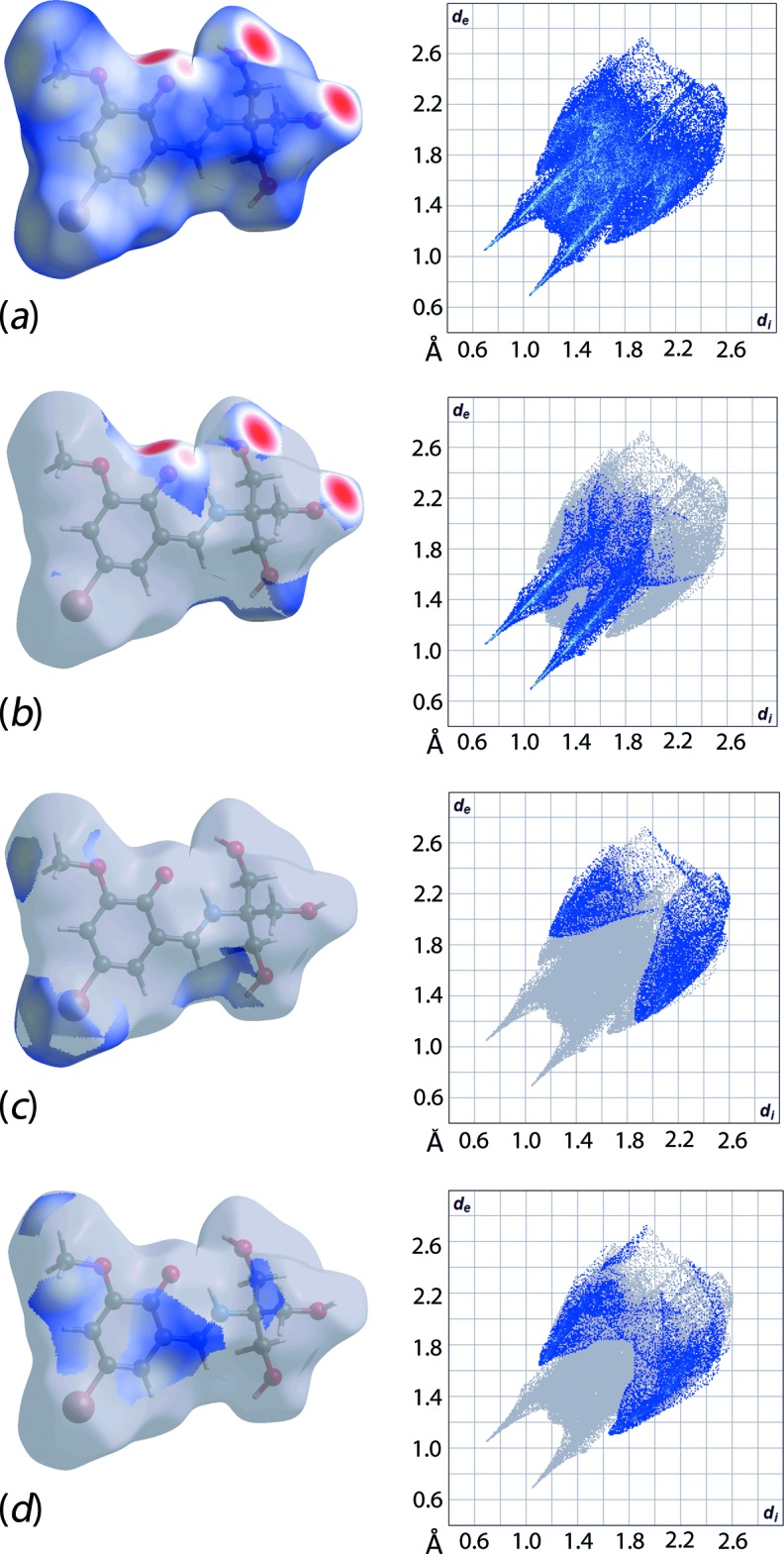
(*a*) Overall Hirshfeld surface and the two-dimensional fingerprint plot for (I), and *d*_norm_ surfaces and two-dimensional plots associated with (*b*) O⋯H/H⋯O, (*c*) Br⋯H/H⋯Br and (*d*) C⋯H/H⋯C inter­actions.

**Figure 4:**
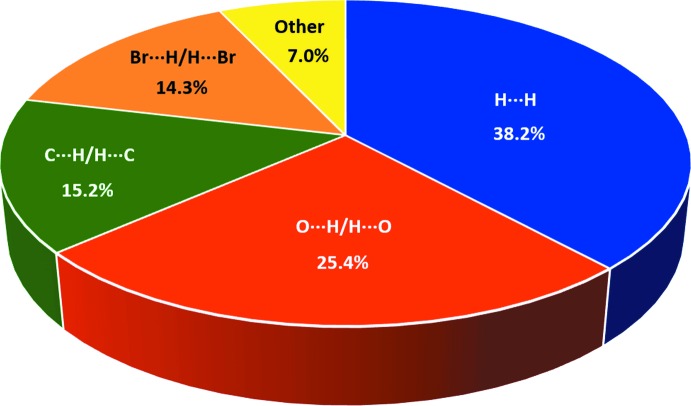
Percentage distribution of the corresponding close contacts to the Hirshfeld surface of (I).

**Figure 5:**
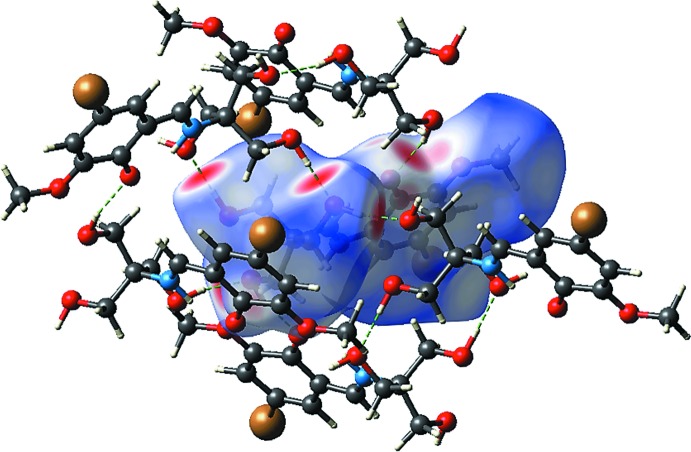
The *d*_norm_ surface for (I), highlighting the O⋯H hydrogen-bonding inter­actions which connect mol­ecules in the mol­ecular packing.

The Hirshfeld surface map provides a visual summary of any close contacts (shown as red) in contrast to relatively long contacts (shown as white and blue). As displayed in Fig. 3 ▸(*a*), there are several red spots observed on the Hirshfeld surface of (I), particularly around the O atoms, indicating close inter­actions at distances shorter than the sum of the van der Waals radii. A qu­anti­tative analysis of the decomposed two-dimensional fingerprint plot of the relevant O⋯H/H⋯O inter­actions reveals a distinctive reciprocal spike in the plot of *d*_e_ *versus d*_i_ (Fig. 3 ▸ *b*), with the sum of contact distances being approximately 1.74 Å, signifying a strong inter­molecular inter­action. Such strong inter­actions constitute the second major contribution to the Hirshfeld surface, *i.e*. 25.4%, between the most prominent H⋯H (38.2%) and other major contacts, like C⋯H/H⋯C (15.2%) and Br⋯H/H⋯Br (14.3%) (Fig. 4 ▸). Their contributions to the overall Hirshfeld surface notwithstanding, as seen from Figs. 3 ▸(*c*) and 3(*d*), C⋯H and Br⋯H contacts are at distances greater than their respective van der Waals radii. Fig. 5 ▸ shows the O—H⋯O inter­actions formed between a reference mol­ecule and symmetry-related mol­ecules.

**Figure 6:**
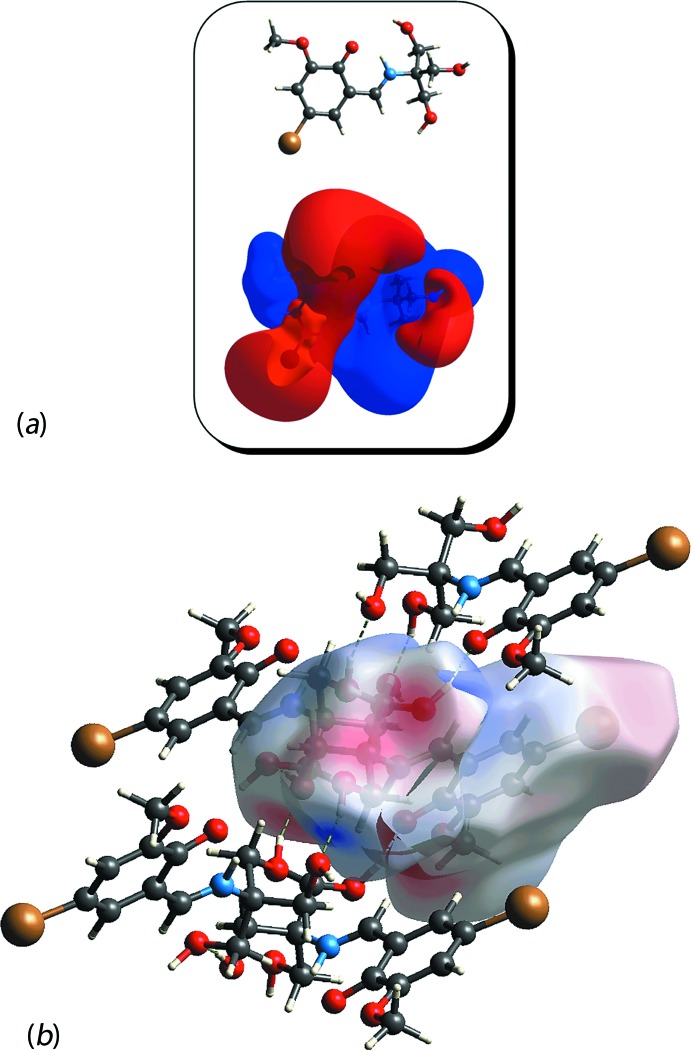
(*a*) The electrostatic potential map of (I) within the range of −0.008 to 0.008 au and (*b*) the ESP mapped over the Hirshfeld surface, showing the attraction between the electronegative (red) and electropositive (blue) sites in (I).

In order to gain a qualitative insight into the electrostatic inter­action and rationalize the packing motif of the structure, the ESP was mapped over the Hirshfeld surface. The result illustrated in Fig. 6 ▸(*a*), shows that the electronegative sites are predominantly converged on O atoms and that, upon crystallization, the electronegative and electropositive sites are connected (Fig. 6 ▸ *b*). It is noteworthy that despite bromine being an electrophilic element, it did not form a significant non-covalent inter­action with neighbouring mol­ecules in the inter-layer region where these atoms are directed. The closest contact in this region occurs with methyl-C⋯H12*C* ^i^, at 3.12 Å, *i.e* beyond the sum of the respective van der Waals radii (Spek, 2009 ▸) [symmetry code: (i) *x*, −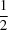 − *y*, 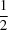 + *z*].

## Database survey   

**Figure 7:**
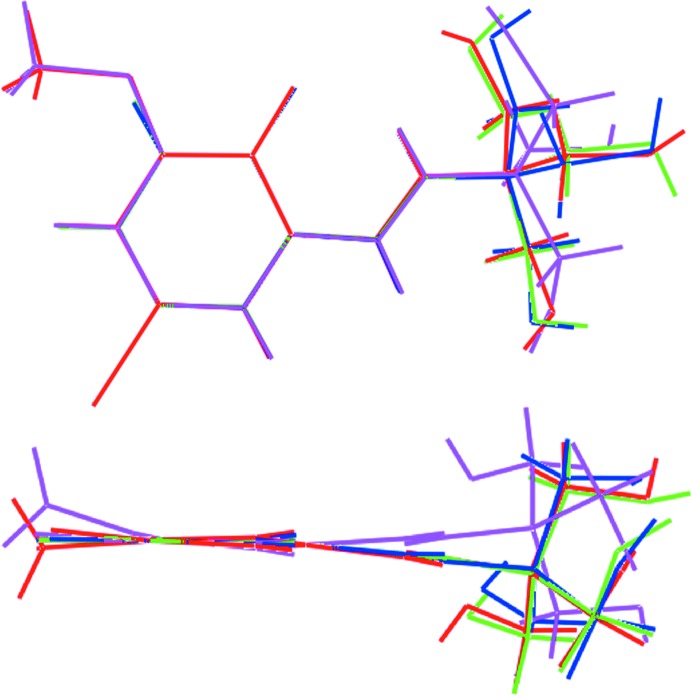
Overlay diagrams for (I) (red image), (II) (green), (III) (blue) and (IV) (pink). Images have been drawn so the benzene rings overlap.

There are several closely related structures to (I) in the crystallographic literature (Groom *et al.*, 2016 ▸). What might be termed the parent compound, *i.e*. with no substitution at the phenolate ring other than the imino group in the 2-position, (II), exists in the keto–amine tautomeric form and has been the subject of several investigations (Asgedom *et al.*, 1996 ▸; Tatar *et al.*, 2005 ▸). Similar zwitterionic structures are found in the 4-bromo, (III) (Martinez *et al.*, 2011 ▸), and 6-meth­oxy, (IV) (Odabas˛oǧlu *et al.*, 2003 ▸), derivatives, both closely related to (I), suggesting this is the most stable form for these mol­ecules, at least in the solid state. Despite the similar electronic structures, conformational differences exist about the ring between (I) and (IV) as seen in the relative dispositions of the meth­oxy groups, *i.e*. C12—O5—C3—C2 is 177.7 (2)° in (I) but −165.75 (14)° in (IV) (Fig. 7 ▸). Differences in conformation of the methyl­hydroxy groups are also apparent, no doubt due to the different hydrogen-bonding patterns in the respective crystal structures.

## Synthesis and crystallization   

A solution of tris­(hy­droxy­meth­yl)amino­methane (1.21 g, 0.01 mol) was added to an ethano­lic solution of 5-bromo-3-meth­oxy-2-hy­droxy­benzaldehyde (2.31 g, 0.01 mol) and refluxed for 2 h. The solution was allowed to stand at room temperature, during which an orange solid formed. This was recrystallized by slow evaporation of its ethanol solution. Yield: 2.67 (80%). Yellow crystals. M.p. 465–466 K. Analysis calculated for C_12_H_16_BrNO_5_: C 44.48, H 3.70, N 1.99%; found: C 44.81, H 3.42, N 1.64%. IR (cm^−1^): 3330 (*b*) ν(N—H, O—H), 1640 (*s*) ν(C=N), 1528 (*m*) ν(—O—C=C—), 1066 (*m*) ν(C—O—C). ^1^H NMR (400 MHz, CDCl_3_): δ 8.35 [*s*, 1H, –N=C(H)], 7.01–7.10 (*m*, 1H, aryl H), 6.83–6.89 (*m*, 1H, aryl H), 5.06 (*s*, 3H, OH), 3.95 (*s*, 3H, OCH_3_), 3.37–3.75 (*m*, 6H, aliphatic H).

## Refinement   

Crystal data, data collection and structure refinement details are summarized in Table 2 ▸. The carbon-bound H atoms were placed in calculated positions (C—H = 0.93–0.97 Å) and were included in the refinement in the riding-model approximation, with *U*_iso_(H) set at 1.2–1.5*U*_eq_(C). The O- and N-bound H atoms were located from difference Fourier maps and refined with distance restraints O—H = 0.82±0.01 Å and N—H = 0.86±0.01 Å, and with *U*_iso_(H) set at 1.5*U*_eq_(O) and *U*_iso_(H) set at 1.2*U*_eq_(N), respectively. Owing to poor agreement, several reflections, *i.e*. (−9 7 7), (−12 4 6), (−10 5 6) and (−3 3 2), were omitted from the final cycles of refinement.

## Supplementary Material

Crystal structure: contains datablock(s) I, global. DOI: 10.1107/S2056989016012159/hb7605sup1.cif

Structure factors: contains datablock(s) I. DOI: 10.1107/S2056989016012159/hb7605Isup2.hkl

CCDC reference: 1496206

Additional supporting information: crystallographic information; 3D view; checkCIF report

## Figures and Tables

**Table 1:** Hydrogen-bond geometry (Å, °)

*D*—H⋯*A*	*D*—H	H⋯*A*	*D*⋯*A*	*D*—H⋯*A*
N1—H1N⋯O1	0.85 (2)	1.90 (2)	2.608 (3)	140 (3)
O2—H2O⋯O4^i^	0.82 (2)	1.93 (2)	2.741 (3)	170 (3)
O3—H3O⋯O2^ii^	0.81 (2)	1.91 (2)	2.704 (3)	167 (4)
O4—H4O⋯O1^iii^	0.82 (3)	1.98 (3)	2.760 (3)	158 (3)
C7—H7⋯O1^iii^	0.93	2.55	3.429 (4)	158
C9—H9*B*⋯O3^i^	0.97	2.51	3.242 (4)	132
C11—H11*B*⋯O1^iv^	0.97	2.39	3.353 (3)	171

Symmetry codes: (i) 
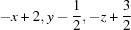
; (ii) 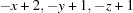; (iii) 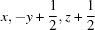; (iv) 
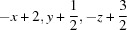
.

**Table 2:** Experimental details

Crystal data	
Chemical formula	C_12_H_16_BrNO_5_
*M*_r_	334.17
Crystal system, space group	Monoclinic, *P*2_1_/*c*
Temperature (K)	293
*a*, *b*, *c* (Å)	12.2872 (9), 10.7186 (8), 10.5830 (8)
β (°)	108.462 (1)
*V* (Å^3^)	1322.06 (17)
*Z*	4
Radiation type	Mo *K*α
μ (mm^−1^)	3.13
Crystal size (mm)	0.26 × 0.10 × 0.08

Data collection	
Diffractometer	Bruker SMART APEX
Absorption correction	Multi-scan (*SADABS*; Sheldrick, 1996 ▸)
*T*_min_, *T*_max_	0.497, 0.788
No. of measured, independent and observed [*I* > 2σ(*I*)] reflections	5095, 2257, 1923
*R*_int_	0.032
(sin θ/λ)_max_ (Å^−1^)	0.595

Refinement	
*R*[*F* ^2^ > 2σ(*F* ^2^)], *wR*(*F* ^2^), *S*	0.030, 0.068, 1.04
No. of reflections	2257
No. of parameters	185
No. of restraints	4
Δρ_max_, Δρ_min_ (e Å^−3^)	0.41, −0.54

Computer programs: *SMART* and *SAINT* (Bruker, 2008 ▸), *SHELXS97* (Sheldrick, 2008 ▸), *SHELXL2014* (Sheldrick, 2015 ▸), *ORTEP-3 for Windows* (Farrugia, 2012 ▸), *QMol* (Gans & Shalloway, 2001 ▸), *DIAMOND* (Brandenburg, 2006 ▸) and *publCIF* (Westrip, 2010 ▸).

## supplementary crystallographic information

### Crystal data

**Table:**

C_12_H_16_BrNO_5_	*F*(000) = 680
*M**_r_* = 334.17	*D*_x_ = 1.679 Mg m^−^^3^
Monoclinic, *P*2_1_/*c*	Mo *K*α radiation, λ = 0.71073 Å
*a* = 12.2872 (9) Å	Cell parameters from 1493 reflections
*b* = 10.7186 (8) Å	θ = 2.6–27.9°
*c* = 10.5830 (8) Å	µ = 3.13 mm^−^^1^
β = 108.462 (1)°	*T* = 293 K
*V* = 1322.06 (17) Å^3^	Prism, yellow
*Z* = 4	0.26 × 0.10 × 0.08 mm

### Data collection

**Table:**

Bruker SMART APEX diffractometer	2257 independent reflections
Radiation source: fine-focus sealed tube	1923 reflections with *I* > 2σ(*I*)
Graphite monochromator	*R*_int_ = 0.032
φ and ω scans	θ_max_ = 25.0°, θ_min_ = 1.8°
Absorption correction: multi-scan (SADABS; Sheldrick, 1996)	*h* = −10→14
*T*_min_ = 0.497, *T*_max_ = 0.788	*k* = −12→12
5095 measured reflections	*l* = −9→12

### Refinement

**Table:**

Refinement on *F*^2^	4 restraints
Least-squares matrix: full	Hydrogen site location: mixed
*R*[*F*^2^ > 2σ(*F*^2^)] = 0.030	*w* = 1/[σ^2^(*F*_o_^2^) + (0.0206*P*)^2^ + 0.8903*P*] where *P* = (*F*_o_^2^ + 2*F*_c_^2^)/3
*wR*(*F*^2^) = 0.068	(Δ/σ)_max_ < 0.001
*S* = 1.04	Δρ_max_ = 0.41 e Å^−^^3^
2257 reflections	Δρ_min_ = −0.54 e Å^−^^3^
185 parameters	

### Special details

**Table:**

Geometry. All esds (except the esd in the dihedral angle between two l.s. planes) are estimated using the full covariance matrix. The cell esds are taken into account individually in the estimation of esds in distances, angles and torsion angles; correlations between esds in cell parameters are only used when they are defined by crystal symmetry. An approximate (isotropic) treatment of cell esds is used for estimating esds involving l.s. planes.

### Fractional atomic coordinates and isotropic or equivalent isotropic displacement parameters (Å^2^)

**Table:**

	*x*	*y*	*z*	*U*_iso_*/*U*_eq_	
Br1	0.45516 (2)	−0.01276 (3)	0.80991 (3)	0.01835 (11)	
O1	0.79706 (16)	0.09687 (18)	0.52558 (18)	0.0134 (4)	
O2	1.05037 (18)	0.27428 (18)	0.60436 (19)	0.0141 (5)	
H2O	1.083 (2)	0.2087 (17)	0.632 (3)	0.021*	
O3	0.96322 (18)	0.60240 (19)	0.62300 (19)	0.0174 (5)	
H3O	0.948 (3)	0.641 (3)	0.5535 (19)	0.026*	
O4	0.85256 (17)	0.54263 (18)	0.83553 (19)	0.0132 (4)	
H4O	0.824 (3)	0.517 (3)	0.891 (2)	0.020*	
O5	0.70096 (17)	−0.12504 (18)	0.49502 (19)	0.0153 (5)	
N1	0.8572 (2)	0.2954 (2)	0.6738 (2)	0.0118 (5)	
H1N	0.865 (3)	0.245 (2)	0.615 (2)	0.014*	
C1	0.7122 (2)	0.1554 (3)	0.6915 (3)	0.0125 (6)	
C2	0.7298 (2)	0.0719 (3)	0.5940 (3)	0.0110 (6)	
C3	0.6701 (2)	−0.0452 (3)	0.5783 (3)	0.0122 (6)	
C4	0.5921 (2)	−0.0711 (3)	0.6427 (3)	0.0133 (6)	
H4	0.5531	−0.1467	0.6289	0.016*	
C5	0.5715 (2)	0.0190 (3)	0.7308 (3)	0.0146 (6)	
C6	0.6309 (2)	0.1276 (3)	0.7580 (3)	0.0140 (6)	
H6	0.6186	0.1835	0.8193	0.017*	
C7	0.7801 (2)	0.2645 (3)	0.7279 (3)	0.0109 (6)	
H7	0.7689	0.3161	0.7933	0.013*	
C8	0.9361 (2)	0.4026 (3)	0.7066 (3)	0.0110 (6)	
C9	1.0558 (2)	0.3539 (3)	0.7151 (3)	0.0129 (6)	
H9A	1.1060	0.4239	0.7159	0.016*	
H9B	1.0878	0.3079	0.7975	0.016*	
C10	0.8904 (2)	0.4963 (2)	0.5932 (3)	0.0120 (6)	
H10A	0.8904	0.4597	0.5094	0.014*	
H10B	0.8124	0.5201	0.5856	0.014*	
C11	0.9462 (2)	0.4610 (3)	0.8413 (3)	0.0121 (6)	
H11A	0.9492	0.3952	0.9052	0.014*	
H11B	1.0175	0.5076	0.8725	0.014*	
C12	0.6500 (3)	−0.2459 (3)	0.4763 (3)	0.0216 (7)	
H12A	0.6684	−0.2879	0.5606	0.032*	
H12B	0.6791	−0.2933	0.4170	0.032*	
H12C	0.5683	−0.2380	0.4388	0.032*	

### Atomic displacement parameters (Å^2^)

**Table:**

	*U*^11^	*U*^22^	*U*^33^	*U*^12^	*U*^13^	*U*^23^
Br1	0.01640 (17)	0.01891 (18)	0.02421 (18)	−0.00122 (13)	0.01279 (13)	0.00285 (13)
O1	0.0139 (11)	0.0159 (11)	0.0136 (10)	−0.0018 (8)	0.0089 (9)	−0.0016 (8)
O2	0.0205 (12)	0.0093 (10)	0.0139 (11)	0.0039 (9)	0.0075 (9)	0.0016 (8)
O3	0.0284 (13)	0.0112 (11)	0.0141 (11)	−0.0034 (9)	0.0091 (10)	0.0029 (8)
O4	0.0141 (11)	0.0153 (11)	0.0145 (11)	0.0004 (8)	0.0108 (9)	−0.0009 (9)
O5	0.0192 (12)	0.0123 (10)	0.0173 (11)	−0.0026 (9)	0.0099 (9)	−0.0049 (9)
N1	0.0151 (13)	0.0091 (12)	0.0109 (13)	−0.0001 (10)	0.0034 (11)	−0.0023 (10)
C1	0.0106 (15)	0.0127 (15)	0.0145 (15)	−0.0002 (12)	0.0043 (12)	0.0015 (12)
C2	0.0079 (14)	0.0123 (15)	0.0110 (14)	0.0025 (11)	0.0008 (12)	0.0037 (12)
C3	0.0094 (15)	0.0149 (15)	0.0126 (15)	0.0007 (12)	0.0038 (12)	−0.0008 (12)
C4	0.0132 (16)	0.0104 (14)	0.0142 (15)	−0.0009 (12)	0.0013 (12)	0.0009 (12)
C5	0.0122 (15)	0.0180 (16)	0.0147 (15)	−0.0013 (12)	0.0060 (12)	0.0054 (12)
C6	0.0140 (15)	0.0140 (15)	0.0145 (15)	0.0035 (12)	0.0052 (12)	0.0007 (12)
C7	0.0121 (15)	0.0098 (14)	0.0115 (14)	0.0028 (11)	0.0049 (12)	0.0025 (11)
C8	0.0124 (15)	0.0105 (14)	0.0114 (14)	−0.0008 (11)	0.0055 (12)	−0.0004 (11)
C9	0.0137 (16)	0.0124 (15)	0.0137 (15)	−0.0019 (12)	0.0056 (12)	−0.0010 (11)
C10	0.0132 (14)	0.0117 (14)	0.0118 (14)	0.0021 (12)	0.0052 (11)	−0.0028 (12)
C11	0.0123 (15)	0.0117 (14)	0.0128 (15)	0.0016 (12)	0.0048 (12)	−0.0002 (11)
C12	0.0263 (19)	0.0138 (16)	0.0267 (18)	−0.0091 (13)	0.0112 (15)	−0.0070 (13)

### Geometric parameters (Å, º)

**Table:**

Br1—C5	1.902 (3)	C4—C5	1.420 (4)
O1—C2	1.287 (3)	C4—H4	0.9300
O2—C9	1.434 (3)	C5—C6	1.355 (4)
O2—H2O	0.818 (10)	C6—H6	0.9300
O3—C10	1.419 (3)	C7—H7	0.9300
O3—H3O	0.815 (10)	C8—C11	1.525 (4)
O4—C11	1.432 (3)	C8—C10	1.529 (4)
O4—H4O	0.819 (10)	C8—C9	1.536 (4)
O5—C3	1.365 (3)	C9—H9A	0.9700
O5—C12	1.425 (3)	C9—H9B	0.9700
N1—C7	1.295 (4)	C10—H10A	0.9700
N1—C8	1.473 (4)	C10—H10B	0.9700
N1—H1N	0.856 (10)	C11—H11A	0.9700
C1—C7	1.417 (4)	C11—H11B	0.9700
C1—C6	1.424 (4)	C12—H12A	0.9600
C1—C2	1.432 (4)	C12—H12B	0.9600
C2—C3	1.438 (4)	C12—H12C	0.9600
C3—C4	1.369 (4)		
			
C9—O2—H2O	109 (2)	N1—C8—C10	106.1 (2)
C10—O3—H3O	105 (2)	C11—C8—C10	111.4 (2)
C11—O4—H4O	107 (2)	N1—C8—C9	107.2 (2)
C3—O5—C12	117.3 (2)	C11—C8—C9	107.0 (2)
C7—N1—C8	127.9 (2)	C10—C8—C9	112.1 (2)
C7—N1—H1N	115 (2)	O2—C9—C8	111.0 (2)
C8—N1—H1N	117 (2)	O2—C9—H9A	109.4
C7—C1—C6	118.9 (3)	C8—C9—H9A	109.4
C7—C1—C2	120.1 (3)	O2—C9—H9B	109.4
C6—C1—C2	121.0 (3)	C8—C9—H9B	109.4
O1—C2—C1	123.0 (3)	H9A—C9—H9B	108.0
O1—C2—C3	120.8 (3)	O3—C10—C8	107.6 (2)
C1—C2—C3	116.2 (3)	O3—C10—H10A	110.2
O5—C3—C4	125.2 (3)	C8—C10—H10A	110.2
O5—C3—C2	112.7 (2)	O3—C10—H10B	110.2
C4—C3—C2	122.1 (3)	C8—C10—H10B	110.2
C3—C4—C5	119.2 (3)	H10A—C10—H10B	108.5
C3—C4—H4	120.4	O4—C11—C8	112.6 (2)
C5—C4—H4	120.4	O4—C11—H11A	109.1
C6—C5—C4	121.8 (3)	C8—C11—H11A	109.1
C6—C5—Br1	119.3 (2)	O4—C11—H11B	109.1
C4—C5—Br1	118.8 (2)	C8—C11—H11B	109.1
C5—C6—C1	119.3 (3)	H11A—C11—H11B	107.8
C5—C6—H6	120.3	O5—C12—H12A	109.5
C1—C6—H6	120.3	O5—C12—H12B	109.5
N1—C7—C1	122.7 (3)	H12A—C12—H12B	109.5
N1—C7—H7	118.6	O5—C12—H12C	109.5
C1—C7—H7	118.6	H12A—C12—H12C	109.5
N1—C8—C11	113.2 (2)	H12B—C12—H12C	109.5
			
C7—C1—C2—O1	−7.6 (4)	C2—C1—C6—C5	1.7 (4)
C6—C1—C2—O1	175.5 (2)	C8—N1—C7—C1	−177.2 (3)
C7—C1—C2—C3	170.7 (2)	C6—C1—C7—N1	178.9 (3)
C6—C1—C2—C3	−6.2 (4)	C2—C1—C7—N1	1.9 (4)
C12—O5—C3—C4	−1.6 (4)	C7—N1—C8—C11	16.6 (4)
C12—O5—C3—C2	177.7 (2)	C7—N1—C8—C10	−105.8 (3)
O1—C2—C3—O5	5.3 (4)	C7—N1—C8—C9	134.3 (3)
C1—C2—C3—O5	−173.1 (2)	N1—C8—C9—O2	45.9 (3)
O1—C2—C3—C4	−175.4 (3)	C11—C8—C9—O2	167.6 (2)
C1—C2—C3—C4	6.2 (4)	C10—C8—C9—O2	−70.0 (3)
O5—C3—C4—C5	177.5 (3)	N1—C8—C10—O3	178.8 (2)
C2—C3—C4—C5	−1.8 (4)	C11—C8—C10—O3	55.2 (3)
C3—C4—C5—C6	−3.2 (4)	C9—C8—C10—O3	−64.6 (3)
C3—C4—C5—Br1	175.5 (2)	N1—C8—C11—O4	−80.2 (3)
C4—C5—C6—C1	3.2 (4)	C10—C8—C11—O4	39.2 (3)
Br1—C5—C6—C1	−175.5 (2)	C9—C8—C11—O4	162.0 (2)
C7—C1—C6—C5	−175.2 (3)		

### Hydrogen-bond geometry (Å, º)

**Table:**

*D*—H···*A*	*D*—H	H···*A*	*D*···*A*	*D*—H···*A*
N1—H1*N*···O1	0.85 (2)	1.90 (2)	2.608 (3)	140 (3)
O2—H2*O*···O4^i^	0.82 (2)	1.93 (2)	2.741 (3)	170 (3)
O3—H3*O*···O2^ii^	0.81 (2)	1.91 (2)	2.704 (3)	167 (4)
O4—H4*O*···O1^iii^	0.82 (3)	1.98 (3)	2.760 (3)	158 (3)
C7—H7···O1^iii^	0.93	2.55	3.429 (4)	158
C9—H9*B*···O3^i^	0.97	2.51	3.242 (4)	132
C11—H11*B*···O1^iv^	0.97	2.39	3.353 (3)	171

Symmetry codes: (i) −*x*+2, *y*−1/2, −*z*+3/2; (ii) −*x*+2, −*y*+1, −*z*+1; (iii) *x*, −*y*+1/2, *z*+1/2; (iv) −*x*+2, *y*+1/2, −*z*+3/2.

## References

[bb1] Asgedom, G., Sreedhara, A., Kivikoski, J., Valkonen, J., Kolehmainen, E. & Rao, C. P. (1996). *Inorg. Chem* **35**, 5674–5683. 10.1021/ic960061r 11666761

[bb2] Back, D. F., Kopp, C. R., de Oliveira, G. M. & Piquini, P. C. (2012). *Polyhedron*, **36**, 21–29.

[bb3] Brandenburg, K. (2006). *DIAMOND*. Crystal Impact GbR, Bonn, Germany.

[bb4] Bruker (2008). *SMART* and *SAINT*. Bruker AXS Inc., Madison, Wisconsin, USA.

[bb5] Chandrasekhar, V., Dey, A., Mota, A. J. & Colacio, E. (2013). *Inorg. Chem* **52**, 4554–4561. 10.1021/ic400073y 23557586

[bb6] Das, C., Vaidya, S., Gupta, T., Frost, J. M., Righi, M., Brechin, E. K., Affronte, M., Rajaraman, G. & Shanmugam, M. (2015). *Chem. Eur. J* **21**, 15639–15650. 10.1002/chem.201502720 26383786

[bb7] Dey, S. K., Mitra, P. & Mukherjee, A. (2015). *Cryst. Growth Des* **15**, 706–717.

[bb8] Dey, S. K. & Mukherjee, A. (2014). *New J. Chem* **38**, 4985–4995.

[bb9] Farrugia, L. J. (2012). *J. Appl. Cryst.* **45**, 849–854.

[bb10] Gans, J. & Shalloway, D. (2001). *J. Mol. Graph. Model.* **19**, 557–559. 10.1016/s1093-3263(01)00090-0 11552684

[bb11] Groom, C. R., Bruno, I. J., Lightfoot, M. P. & Ward, S. C. (2016). *Acta Cryst.* B**72**, 171–179. 10.1107/S2052520616003954 PMC4822653 27048719

[bb12] Halevas, E., Tsave, O., Yavropoulou, M. P., Hatzidimitriou, A., Yovos, J. G., Psycharis, V., Gabriel, C. & Salifoglou, A. (2015). *J. Inorg. Biochem* **147**, 99–115. 10.1016/j.jinorgbio.2015.03.009 25920352

[bb13] Lee, S. M., Sim, K. S. & Lo, K. M. (2015). *Inorg. Chim. Acta*, **429**, 195–208.

[bb14] Martinez, R. F., Ávalos, M., Babiano, R., Cintas, P., Jiménez, J. L., Light, M. E. & Palacios, J. C. (2011). *Eur. J. Org. Chem*. pp. 3137–3145.

[bb15] McKinnon, J. J., Jayatilaka, D. & Spackman, M. A. (2007). *Chem Commun*. pp. 3814–3816. 10.1039/b704980c 18217656

[bb16] Odabas˛oǧlu, M., Albayrak, Ç., Büyükgüngör, O. & Lönnecke, P. (2003). *Acta Cryst.* C**59**, o616–o619. 10.1107/s0108270103020997 14605408

[bb17] Rehder, D., Pessoa, J. C., Geraldes, C. F. G. C., Castro, M. M. C. A., Kabanos, T., Kiss, T., Meier, B., Micera, G., Pettersson, L., Rangel, M., Salifoglou, A., Turel, I. & Wang, D. (2002). *J. Biol. Inorg. Chem* **7**, 384–396. 10.1007/s00775-001-0311-5 11941496

[bb18] Sheldrick, G. M. (1996). *SADABS*. University of Göttingen, Germany.

[bb19] Sheldrick, G. M. (2008). *Acta Cryst.* A**64**, 112–122. 10.1107/S0108767307043930 18156677

[bb20] Sheldrick, G. M. (2015). *Acta Cryst.* C**71**, 3–8.

[bb21] Spackman, M. A. & Jayatilaka, D. (2009). *CrystEngComm*, **11**, 19–32.

[bb22] Spackman, M. A. & McKinnon, J. J. (2002). *CrystEngComm*, **4**, 378–392.

[bb23] Spackman, M. A., McKinnon, J. J. & Jayatilaka, D. (2008). *CrystEngComm*, **10**, 377–388.

[bb24] Spek, A. L. (2009). *Acta Cryst.* D**65**, 148–155. 10.1107/S090744490804362X PMC2631630 19171970

[bb25] Tatar, L., Nazir, H., Gümüşer, M., Kale, C. & Atakol, O. (2005). *Z. Kristallogr* **220**, 639–642.

[bb26] Tsave, O., Halevas, E., Yavropoulou, M. P., Kosmidis Papadimitriou, A., Yovos, J. G., Hatzidimitriou, A., Gabriel, C., Psycharis, V. & Salifoglou, A. (2015). *J. Inorg. Biochem* **152**, 123-137. 10.1016/j.jinorgbio.2015.08.014 26383120

[bb27] Westrip, S. P. (2010). *J. Appl. Cryst.* **43**, 920–925.

[bb28] Wolff, S. K., Grimwood, D. J., McKinnon, J. J., Turner, M. J., Jayatilaka, D. & Spackman, M. A. (2012). *Crystal Explorer*. The University of Western Australia.

[bb29] Wu, G., Hewitt, I. J., Mameri, S., Lan, Y., Clérac, R., Anson, C. E., Qiu, S. & Powell, A. K. (2007). *Inorg. Chem.* **46**, 7229–7231. 10.1021/ic070296a 17676836

[bb30] Zou, H.-H., Sheng, L.-B., Liang, F. P., Chen, Z.-L. & Zhang, Y.-Q. (2015). *Dalton Trans* **44**, 18544–18552. 10.1039/c5dt03368c 26443303

